# Determinants of personal exposure to PM_2.5_ and black carbon in Chinese adults: A repeated-measures study in villages using solid fuel energy

**DOI:** 10.1016/j.envint.2020.106297

**Published:** 2021-01

**Authors:** Martha Lee, Ellison Carter, Li Yan, Queenie Chan, Paul Elliott, Majid Ezzati, Frank Kelly, James J. Schauer, Yangfeng Wu, Xudong Yang, Liancheng Zhao, Jill Baumgartner

**Affiliations:** a Department of Epidemology, Biostatistics and Occupational Health, McGill University, Montreal, Quebec, Canada; b Institute on the Environment, University of Minnesota, Saint Paul, MN, USA; c Department of Civil and Environmental Engineering, Colorado State University, Fort Collins, CO, USA; e Department of Epidemiology and Biostatistics, School of Public Health, Imperial College London, London, UK; f Department of Analytical, Environmental & Forensic Sciences, School of Population Health and Environmental Sciences, Kings College London, London, UK; g MRC Centre for Environment and Health, School of Public Health, Imperial College London, London, UK; h NIHR Imperial College London Biomedical Research Centre, London, UK; i Department of Civil and Environmental Engineering, University of Wisconsin, Madison, USA; j Environmental Chemistry & Technology Program, University of Wisconsin, Madison, USA; k Peking University Clinical Research Institute, Beijing, China; l Department of Building Science, School of Architecture, Tsinghua University, Beijing, China; m National Center for Cardiovascular Disease, Fuwai Hospital, Peking Union Medical College & Chinese Academy of Medical Sciences, Beijing, China; n Institute for Health and Social Policy, McGill University, Montreal, Canada

**Keywords:** Air pollution, Energy, Household air pollution, Repeated measures

## Abstract

Exposure to air pollution is a leading health risk factor. The variance components and contributions of indoor versus outdoor source determinants of personal exposure to air pollution are poorly understood, especially in settings of household solid fuel use. We conducted a panel study with up to 4 days of repeated measures of integrated gravimetric personal exposure to PM_2.5_ and black carbon in 787 men and women (ages 40–79) living in *peri*-urban villages in northern (Beijing and Shanxi) and southern (Guangxi) China. We simultaneously measured outdoor PM_2.5_ and collected questionnaire data on sociodemographic characteristics and indoor pollution sources including tobacco smoking and solid fuel stove use. We obtained over 2000 days of personal exposure monitoring which showed higher exposures in the heating season (geometric mean (GM): 108 versus 65 μg/m^3^ in the non-heating season for PM_2.5_) and among northern participants (GM: 90 versus 59 μg/m^3^ in southern China in the non-heating season for PM_2.5_). We used mixed-effects models to estimate within- and between-participant variance components and to assess the determinants of exposures. Within-participant variance in exposure dominated the total variability (68–95%). Outdoor PM_2.5_ was the dominant variable for explaining within-participant variance in exposure to PM_2.5_ (16%). Household fuel use (PM_2.5_: 8%; black carbon: 10%) and smoking status (PM_2.5_: 27%; black carbon: 5%) explained the most between-participant variance. Indoor sources (solid fuel stoves, tobacco smoking) were associated with 13–30% higher exposures to air pollution and each 10 μg/m^3^ increase in outdoor PM_2.5_ was associated with 6–8% higher exposure. Our findings indicate that repeated measurements of daily exposure are likely needed to capture longer-term exposures in settings of household solid fuel use, even within a single season, and that reducing air pollution from both outdoor and indoor sources is likely needed to achieve measurable reductions in exposures to air pollution.

## Highlights

• PM_2.5_ exposures were on average 2 times higher than outdoor PM_2.5_.
• Men and women had similar exposures to PM_2.5_ after accounting for smoking.
• Within-individual variances were much larger than between-individual variances.
• Indoor sources explained 7–28% of between-individual variance in PM_2.5_ exposure.
• Outdoor PM_2.5_ explained 16% of within-individual variance in PM_2.5_ exposure.

## 1 Introduction

Air pollution is a leading global concern for human health (Health Effects Institute, 2019). Exposure to fine particulate matter (PM_2.5_) air pollution is independently associated with the development of cardio-respiratory diseases and other adverse health outcomes throughout the life course including low birth weight and neurocognitive outcomes (Bourdrel et al., 2017, Health Effects Institute, 2019). Air pollution ranks as the 5th leading risk factor for global mortality, responsible for an estimated 4.9 million premature deaths in 2017 (Health Effects Institute, 2019). Low- and middle-income countries comprise a substantial share of this burden, accounting for over 90% of PM_2.5_-attributable deaths (Health Effects Institute, 2019).

The source contributors to air pollution are diverse, even in rural and *peri*-urban settings (Secrest et al., 2017). Outdoor emissions sources like traffic, industry, and agricultural burning are large contributors to PM_2.5_ in these settings (Karagulian et al., 2015, Liao et al., 2017). Indoor sources like tobacco smoking and household use of solid fuel stoves (used in 47% of homes globally for cooking) emit high levels of PM_2.5_ into homes and communities (Health Effects Institute, 2019). The relative contribution of indoor versus outdoor sources to exposures to PM_2.5_ is poorly understood, particularly in low and middle-income countries, in large part due to the relatively few studies with measured personal exposures (Carter et al., 2017). Understanding the determinants of exposure has important implications for air pollution interventions and policies. Recent intervention studies, for example, hypothesized that pollution from traffic and poor outdoor air quality limited the effectiveness of household stove interventions in measurably reducing exposures to PM_2.5_ (Secrest et al., 2017, Yip et al., 2017, Pilishvili et al., 2016, Pope et al., 2017, Liu et al., 2018).

Although an increasing number of studies have measured personal exposures to PM_2.5_ in settings of solid fuel burning (Shupler et al., 2018), very few have included repeated measures of exposure (McCracken et al., 2009, Arku et al., 2015, Baumgartner et al., 2019, Sanchez et al., 2019, Baumgartner et al., 2011). Instead, most studies involved a single short-term (24- or 48-h) measurement (Carter et al., 2017) and focused on PM_2.5_ mass but were unable to evaluate specific components of PM that could indicate its toxicity (e.g., black carbon). This limits our understanding of how to best assess ‘usual’ exposure to air pollution in settings of household solid fuel use, which is the metric most relevant for epidemiologic and intervention studies. There is also a lack of air pollution exposure data for important population subgroups. Men, for example, account for nearly half of the modelled disease burden attributable to household air pollution (Institute for Health Metrics and Evaluation, 2020), but few studies have measured men’s exposure to PM_2.5_ in a setting where solid fuel stoves were used (Sanchez et al., 2019, Arku et al., 2018, Shupler et al., 2020). Measurements of PM_2.5_ exposures in exclusive clean fuel users relative to users of solid fuel in the same setting are rare, which is important for more realistically estimating the potential air quality and health benefits of clean energy interventions (Shupler et al., 2018, Shupler et al., 2020).

Leveraging 2246 measurement days of personal exposure to PM_2.5_ and black carbon from 787 participants enrolled in the INTERMAP China Prospective (ICP) study, this study aims to 1) characterize the levels and seasonal patterns of air pollution exposures for men and women living in northern and southern China, 2) describe the variability in exposures within- and between-participants, and 3) evaluate the contribution of indoor and outdoor sources of air pollution to personal exposures.

## 2 Methods

### 2.1 Study design and population

The ICP study design and population are described in detail elsewhere (Yan et al., 2019). In brief, 787 adults (ages 40–79, 55% female) from 17 villages in three provinces in northern (Beijing and Shanxi) and southern (Guangxi) China were enrolled into the study in 2015 and 2016 (Supplementary Fig. S1). These regions were selected for study because of their diversity in geography and environmental risk factors for disease, including household fuel use. Coal fuel is commonly used for residential heating in northern China and is a large contributor to household and outdoor air pollution (Health Effects Institute, 2019, Liao et al., 2017), whereas the southern province of Guangxi is sub-tropical and does not have a distinct heating season. Biomass (i.e., wood and crop residues) stoves are used for cooking in all three sites, often alongside low-polluting electric and gas-powered stoves. Detailed information on household energy use practices in our study homes is published elsewhere (Carter et al., 2019).

Most ICP study participants were originally enrolled into the International Study of Macro/Micronutrients and Blood Pressure (INTERMAP), a cross-sectional study which randomly selected households in the study villages between 1995 and 1997, and then randomly selected one adult from each household to participate. We re-enrolled 575 of the 680 surviving INTERMAP participants (85% participation rate) into the ICP study (ages 60–79; 53% female), in addition to 212 adults (88% participate rate) ages 40–59 that were randomly selected from the same villages to evaluate cohort differences in environmental risk factors over time. We obtained written informed consent from all participants. Ethical approvals were obtained from all investigator institutions (McGill: #A08-M37-16B; Fu Wai Hospital: #2015–650; Imperial: #15IC3095, Peking: #00001052–15017, Tsinghua: #20140077).

### 2.2 Data collection

Measurement campaigns were conducted in Shanxi in August 2015 and November 2015; Beijing in December 2015 and September 2016; and in Guangxi in November 2016. We conducted two campaigns in the northern sites to capture the heating and non-heating seasons, which can impact household energy use and air pollution exposures (Carter et al., 2017).

For data collection, participants travelled to clinics that were centrally located in their villages, typically by foot or electric bicycle. Trained staff carried out the study measurements using the same standardized procedures across all sites (Yan et al., 2019). At the first clinic visit in each campaign, participants were fitted with personal air monitors and completed questionnaires on individual and household characteristics including energy use. Participants returned to the clinic after 24-h to exchange the air monitors for new ones and returned again after a second 24-h period to return their monitors. Staff conducted home visits if participants was unable to travel to the clinics. Outdoor air quality and ambient temperature were measured throughout the campaigns. Descriptions of these study measurements are summarized below and detailed information is published elsewhere (Yan et al., 2019).

#### 2.2.1 Personal exposure to PM_2.5_

We obtained 2246 measurements of integrated 24-h exposure to PM_2.5_ using Harvard Personal Exposure Monitors (H-PEM) (Mesa Labs, USA) that housed Zefluor^TM^ 37 mm PTFE filters (Pall Life Sciences, USA) and were attached downstream from a personal sampling pumps (Apex Pro and TUFF™, Casella Inc; USA) operated at 1.8 L/min (Demokritou et al., 2001). Air monitors were placed inside waistpacks that participants were asked to wear at all times possible and to keep within 2 m while sleeping, sitting, or bathing (Supplementary Fig. S2). In a subsample of exposure measurements (n = 1595, 76% of all measurements), we added a pedometer (HJ-321 Tri-Axis, Omron; Japan) to the waistpack to monitor compliance in wearing them. Participants with 24-h step counts of < 500 steps were considered potentially non-compliant in wearing the air monitor on that day (n = 47, 3%).

Pump flow rates were measured at the start and end of each sampling period using a rotameter that was field calibrated at the beginning and middle of each measurement campaign using a primary gas flow standard (mini-BUCK Calibrator M−5; A.P. Buck Inc.; Orlando, FL, USA). For quality control and to address potential contamination, we collected ~ 7% field blank filters that were placed inside identical H-PEMs and cyclones, subject to the same field conditions, and analyzed using the same protocol as the filter samples.

#### 2.2.2 Outdoor PM_2.5_

We obtained real-time outdoor PM_2.5_ measurements for our study period from nearby government air monitoring stations equipped with reference-quality monitors (i.e., tapered element oscillating microbalance (TEOM); (http://beijingair.sinaapp.com). Hourly data from all stations within 50 km of the village centers were inverse distance weighted (power function of 1) to calculate a mean hourly concentration for each study village. We then calculated 24-h average outdoor PM_2.5_ values that corresponded with the date and time of the personal 24-h exposure measurements. Personal exposure measurements generally started at 10:00am and ended at 10:00am of the next day so outdoor PM_2.5_ averages ran from 10:00am to 10:00am of the next day. We also measured village-level integrated gravimetric PM_2.5_ during one data collection campaign at each study site. The gravimetric monitors were positioned at least 4 m from the ground in a location that was 1) central to each village, 2) at least 30 m from a household chimney, and 3) at least 100 m from other PM_2.5_ emission sources including local industry and major roadways. We placed PTFE filters into either H-PEMs or cyclones (Mesa Laboratories, USA) that were attached to sampling pumps with flow rates of 1.8 or 3.5 lpm, respectively. The filters were collected every 24-h and replaced with new ones. Village-level measurements of PM_2.5_ were highly correlated with values estimated from government sensors on the same day (n = 42 days of paired observations; Pearson *r* = 0.87; RMSE = 45.4) (Supplementary Fig. S3). To quantify outdoor PM_2.5_, we used the village-level measurements when available (34% of study days) and used the estimated PM_2.5_ values for the remaining days.

#### 2.2.3 Laboratory analysis of PTFE filters for PM_2.5_ and black carbon

Gravimetric analysis was used to determine the PM_2.5_ mass on filter samples and blanks. Following at least 24-h of conditioning in a temperature and humidity-controlled environment at the Wisconsin State Hygiene Laboratory (Madison, WI), the filters were weighed in duplicate using a microbalance (MX-5; Mettler-Toledo, Columbus, OH, USA). If the difference between the first two weights exceeded 15 μg, a third measurement was obtained, and the two closest weights were averaged for statistical analysis. The microbalance’s zero and span were checked after every batch of 10 filters. Pre-sampling filter weights were subtracted from the post-sampling weights. The filter mass (μg) was divided by the volume of air (m^3^) pulled through the filter during sampling to calculate the PM_2.5_ concentration.

Black carbon was measured on filters using an Aethalometer (SootScan^TM^ Model OT21 Transmissometer, Magee Scientific; USA). Black carbon is a component of PM_2.5_ and a product of incomplete combustion that may more strongly associated with adverse health outcomes than the mass of PM_2.5_ (Baumgartner et al., 2014, Janssen et al., 2011). The optical method estimates black carbon by evaluating the attenuation of light through the sample and blank PTFE filters compared with that of a reference filter. To equate the optical black carbon measurements to elemental carbon, we applied the U.S. EPA sigma of 4.2 and used an empirical correction factor based on the black carbon-elemental carbon associations in previous air pollution campaigns in rural China that used the same filter media (Baumgartner et al., 2018). Specifically, we applied the linear correction factor of 0.092 with adjusted observed values ranging from 0.0085 to 11.4 μg/m^3^. The corrected black carbon mass loadings (µg/cm^2^) were converted to concentration (µg/m^3^) by multiplying the mass loading (µg/cm^2^) by the area of each filter (9.03 cm^2^), and then dividing the mass by the volume of air sampled (m^3^).

Season-specific blank values for PM_2.5_ and black carbon were calculated for each study site and subtracted from the net filter weights and attenuated infrared values, respectively. We replaced negative blank-corrected values (PM_2.5_: n = 15 filters, <0.01%; black carbon: n = 33 filters, <1%) by randomly assigning a value between 0 and half the limit of detection, which was 4 µg for PM_2.5_ and 0.22 µg/m^3^ for black carbon. We excluded filters from the statistical analysis if they were damaged (n = 3 for PM_2.5_ and n = 1 for black carbon; <0.01% of filter samples); could not be matched to a participant due to data entry errors (n = 21; <0.01%); had net weights that exceeded a realistic 24-h mass, indicating infiltration of larger-sized particles onto the filter, filters being switched, or unseen filter damage (n = 7; <0.01%); or failed to capture at least 10-h of the 24-hr target due to pump failure (n = 108; 4.8%). An unrealistic weight was defined as net weights <0 μg or over 2500 μg. Filters exceeding these weights were flagged and assessed for any abnormalities (e.g., filter damage or visible dust). For the main analysis, we used a 10-h cut-off for completeness because this time period captured most of the daytime hours. Of the 148 samples that ran for <23 h but more than 10 h, 70 ran for 10 to 19.2 h (19.2 h = 80% of the 24-h sampling time), and 78 samples ran between 19.2 and 23 h. The remaining filter-based measurements were considered ‘complete’ and included in the statistical analysis.

#### 2.2.4 Meteorological data

We obtained real-time temperature and dew point temperature data from the U.S. National Oceanic and Atmospheric Administration (https://www.ncdc.noaa.gov/isd/products). Relative humidity was estimated based on the temperature and dew point temperature the using the weathermetrics package in R (Anderson and Peng, 2012). We used inverse distance weighting (power function of 1) from all meteorological stations within 100 km of each study village to estimate the daily temperature and relative humidity. To evaluate the accuracy to these data, we compared them to the outdoor temperatures measured during the participants clinic visits using local meteorological stations (Yan et al., 2019) (n = 99 days; Pearson *r* = 0.97; RMSE = 4.5) (Supplementary Fig. S4). We used the estimated temperatures for statistical analysis because they were highly correlated with measured temperature and also allowed us to time-match meteorological data with exposure measurements.

#### 2.2.5 Questionnaires

Staff administered questionnaires in Mandarin-Chinese to collect information on variables potentially related to energy use and exposures to air pollution including age, gender, ethnicity, education, occupation, marital status, tobacco smoking, and household income. We drew questions from the INTERMAP study that were re-tested with local residents to ensure that questions were being interpreted as intended (Yan et al., 2019). We also collected comprehensive information on household fuels, energy devices, and ventilation using an image-based questionnaire that included pictures of all stoves and fuels used in the region. Detailed information on the energy questionnaire is provided elsewhere (Carter et al., 2019). Briefly, respondents indicated whether they were currently using a given energy device or fuel and, if so, described the frequency and purpose of use. Energy devices that burned coal, wood, and/or agricultural residues were categorized as ‘solid fuel’ stoves, while stoves powered by gas or electricity were considered ‘clean fuel’ stoves. All devices were classified into one of the following categories: solid fuel cookstoves, clean fuel cookstoves, solid fuel heating stoves, and clean fuel heating stove. Participants were categorized as ‘exclusive clean cooking fuel’ users if they reported using clean fuel regularly and reported no use or rare use (i.e., holidays or when hosting guests) of solid fuel. The remaining participants were classified as users of solid fuel for cooking. Solid fuel stove use was further divided into any indoor use or only outdoor use. Heating fuel included the same categories as cooking with the addition of a fourth category to indicate no heating or cooling-specific device in the home. For cooking fuel, outdoor-only solid fuel use and indoor solid fuel use were combined into a single category due to a small sample size.

### 2.3 Statistical analysis

Air pollution summary statistics were calculated by season, study site, gender, and energy use. Pollution exposures exhibited positive skewness, whereas the corresponding natural log-transformed values were approximately normally distributed and were thus used for statistical analyses. We evaluated whether measurement sequence may have systematically impacted exposure using scatterplots and paired t-tests that compared the first and second measurement day for each season and site.

#### 2.3.1 Estimating with-individual and between-individual exposure variability

We used a series of mixed-effects regression models to leverage the repeated measures of air pollution and partition the total variance in exposure into its within-individual and between-individual components. We started with the following base (intercept-only) model:

$$\ln\left(Y_{\mathrm{ik}}\right)=\beta_{0}+b_{i}+\varepsilon_{\mathrm{ik}}$$

where ln*(Y_ik_)* is the *k^th^* measurement of log-transformed pollution (PM_2.5_ or black carbon) for participant *i*, *b_i_* is the participant random effect and *ε_ik_* is the remaining error with variance components of *σ_b_*^2^ and *σ_ε_*^2^, respectively. These can be roughly interpreted as the variance between-individuals (*σ_b_*^2^) and the variance within-individuals (*σ_ε_*^2^). We estimated the intraclass correlation coefficient (ICC; i.e., the proportion of total variability in exposure attributed to between-individual differences) by: *σ_b_*^2^/(*σ_b_*^2^ + *σ_ε_*^2^). These models assume that the *b_i_* and the $\varepsilon_{\mathrm{ik}}$ are independent and normally distributed with variances of *σ_b_*^2^ and *σ_w_*^2^, respectively, and have a compound symmetry correlation structure.

#### 2.3.2 Explaining variability in exposure to PM_2.5_ and black carbon

We evaluated the proportion of each variance component explained by indoor and outdoor sources of air pollution and by other socio-demographic and environmental variables by comparing the base (intercept-only) model to a set of models containing an increasing number of independent variables. We evaluated variables that were determined *a priori* to be associated with exposure to air pollution in past studies (see variables listed in Table 1) (Baumgartner et al., 2011, Ni et al., 2016). We imputed missing data on yearly income for 93 participants (12%) using multiple imputation with the MICE package in R (Sv and Groothuis-Oudshoorn, 2010). Separate models were conducted for exposure to PM_2.5_ and black carbon.

**Table 1:** Characteristics of study participants by study site [n (n%) or mean (standard deviation, sd)].

**Characteristic**	**Guangxi (n = 239)**	**Beijing (n = 258)**	**Shanxi (n = 290)**
**Age (years), mean (sd)**	63.4 (9.4)	63.6 (7.6)	62.0 (8.7)
**Gender**			
female	128 (53.6)	149 (57.8)	157 (54.1)
male	107 (44.8)	108 (41.9)	133 (45.9)
missing	4 (1.7)	1 (0.4)	0
**Ethnicity**			
Han	122 (51.0)	255 (98.8)	290 (100.0)
Zhuang	113 (47.3)	0	0
other	0	2 (0.8)	0
missing	4 (1.7)	1 (0.4)	0
**Occupation**			
subsistence farming	34 (14.2)	200 (77.5)	213 (73.4)
other work outside the home	30 (12.6)	15 (5.8)	21 (7.2)
not working outside the home^a^	171 (71.5)	42 (16.3)	56 (19.3)
missing	4 (1.7)	1 (0.4)	0
**Martial status**			
married/cohabitation	175 (73.2)	229 (88.8)	255 (87.9)
widowed	51 (21.3)	24 (9.3)	31 (10.7)
divorce/separated/unmarried	9 (3.8)	4 (1.6)	4 (1.4)
missing	4 (1.7)	1 (0.4)	0
**Household income in the past year**			
<2000 yuan	29 (12.1)	135 (52.3)	199 (68.6)
≥2000 yuan	206 (86.2)	122 (47.3)	91 (31.4)
missing	4 (1.7)	1 (0.4)	0
**Highest education attained**			
no formal education	29 (12.1)29 (12.1)	61 (23.6)	30 (10.3)
primary school	101 (42.3)	86 (33.3)	137 (47.2)
early high school/college	105 (43.9)	110 (42.6)	123 (42.4)
missing	4 (1.7)	1 (0.4)	0
**Tobacco smoking**			
current smoker	40 (16.7)	56 (21.7)	85 (29.3)
non-smoker w/ household smoker	59 (24.7)	77 (29.8)	72 (24.8)
non-smoker w/o household smoker	136 (56.9)	125 (48.4)	133 (45.9)
missingmissing	4 (1.7)	0	0
**Fuel used for cooking^b^**			
exclusive clean fuel	69 (28.9)	163 (63.2)	129 (44.5)
solid fuel, indoor	154 (64.4)	81 (31.4)	155 (53.4)
solid fuel, outdoor only	1 (0.4)	1 (0.4)	0
missing	15 (2.1)	13 (5.0)	6 (2.1)
**Fuel used for space heating^b^**			
exclusive clean fuel	70 (29.3)	61 (23.6)	88 (30.3)
solid fuel, indoor	0	170 (65.9)	151 (52.1)
solid fuel, outdoor only	0	11 (4.3)	29 (10.0)
no device	154 (64.4)	3 (1.2)	16 (5.5)
missing	15 (2.1)	13 (5.0)	6 (2.1)

a Includes housekeeping, retired, and unemployed.

b Clean fuel includes natural gas, liquified petroleum gas (LPG), and electricity; solid fuel includes coal and biomass. For cooking fuel use, participants were assigned to the following categories: (1) exclusive clean fuel (i.e., use of gas or electricity and no or only rare use of solid fuel (i.e., holidays or when hosting guests); (2) solid fuel, indoor stove (i.e., use of at least 1 solid fuel stove indoors), or (3) solid fuel, outdoor only (i.e., use of solid fuel stove but only outdoors). For heating, we added the additional category of “no device” (i.e., no heating-specific devices in the home).

To assess the models’ explanatory power and the fit of data, the proportion of within-individual variance explained (R^2^_within_) was calculated by subtracting from 1 the ratio of residual within-individual variance under each alternative mixed model to that of the base model, as described elsewhere (Xu, 2003). Between-individual variance explained (R^2^_between_) was calculated in an analogous way. To evaluate the prediction accuracy of these models, we excluded a random 20% subsample of observations to create the appearance of missing data. The remaining data were used to estimate the full model with all covariates and then predict the excluded observations. We ran each model 100 times, each run dropping a different random 20% subset of the data. For each model run, we calculated the root mean square error (RMSE) and Spearman correlation between predicted and measured exposures. The final estimates are the averages of 100 runs.

The linear mixed-effects regression models were conducted in R (R Core Team. R, 2013, version 3.4.2) using the lme function from the nlme package (Pinheiro et al., 2017). Collinearity among the independent variables was investigated using Pearson correlation matrices and variance inflation factors, and the assumptions of normality of residual errors and homoscedasticity were evaluated by graphical analysis of residuals. To assess assumptions of linearity for continuous independent variables, we generated response functions using natural cubic spline models with 2 and 3 degrees of freedom (Wang and Yan, 2017); (R Core Team. R, 2013). All response functions were consistent with a linear association and thus replaced by linear functions. Marginal and conditional R^2^ values (Nakagawa and Schielzeth, 2013) were calculated to compare the results from the PM_2.5_ and black carbon prediction models.

We conducted a number of sensitivity analyses for the PM_2.5_ modelling. We also conducted separate models by gender and season, and limited the analysis to exposure observations where the measurement duration was within ± 10% of the 24-h target (n = 1969; 95% of observations). To assess whether use of outdoor PM_2.5_ from the government monitors versus village-level measurements impacted our results, we restricted the regression analyses to exposure measurements taken on the same day as village-level outdoor PM_2.5_ (n = 619; 30% of observations), and compared those results to models including outdoor PM_2.5_ from government monitors. To assess potential non-compliance wearing personal samplers, we restricted the regression analyses to samples with associated step counts greater than 500 steps.

## 3 Results

### 3.1 Characteristic of the study participants

Participants ranged in age from 40 to 79 years (mean: 63) and were 55% female **(**Table 1**)**. Most participants in the north (Beijing, Shanxi) were subsistence farmers (76%), while most participants in Guangxi were either retired or not working (73%). Exclusive use of clean fuel for cooking (48%) was more common than exclusive use of clean fuel for heating (38% among those reporting space heating). Nearly half (49%) of men were tobacco smokers. Very few women smoked (2%), though 45% of non-smoking women lived with at least one smoker.

### 3.2 Personal exposures to PM_2.5_ and black carbon

We obtained 2073 complete 24-h measurements of personal exposure to PM_2.5_ (92% of attempted), of which 1291 were collected in the non-heating season and 782 in the heating season. Of the 787 study participants, 778 of the 787 participants had at least 1 complete PM_2.5_ measurement and 703 had 2 complete measurements. In the northern sites with 2 seasons of measurements, 370 participants had 3 complete measurements and 223 had 4 complete measurements. Most (97%) post-sampling pump flow rates were within ± 10% of the target flow rate.

Daily (24-h) exposures to PM_2.5_ and black carbon ranged from 0.01 to 1528 and 0.00–12 μg/m^3^, respectively. Overall, 92% of 24-h PM_2.5_ exposure measurements were higher than the World Health Organization (WHO) guideline of 25 μg/m^3^ (88% in Guangxi; 90% in Beijing; 96% in Shanxi), and 79% of exposure measurements were higher than outdoor PM_2.5_ on the same day (68% in Guangxi; 74% in Beijing; 90% in Shanxi). We found low to moderate correlations between exposures to PM_2.5_ and black carbon on the same day (r = 0.49) and between the same pollutant on the first and second measurement days (r = 0.44 for PM_2.5_; r = 0.40 for black carbon), with little difference by season (Supplementary Fig. S5). The correlation between daily personal exposure and outdoor air pollution concentrations from the same day was low (r = 0.33 for PM_2.5_; r = 0.40 for black carbon).

**Fig. 1:**
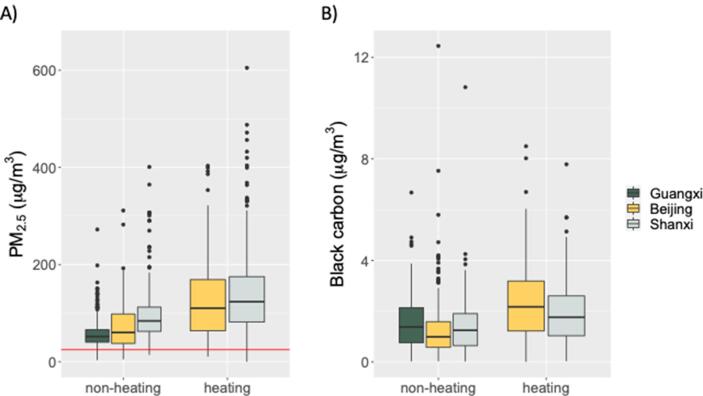
Distributions of average 24-h exposures to A) PM_2.5_ and B) black carbon in *peri*-urban Chinese adults (n = 787), by season and study site^a^. The red line indicates the World Health Organization’s 24-h PM_2.5_ guideline of 25 μg/m^3^. ^a^We averaged repeat exposure samples from the same season so that each participant only contributed one measurement per season. The y-axis for PM_2.5_ was limited to 650 μg/m^3^ to facilitate visual comparison, which excluded 3 observations (710, 838, and 1241 μg/m^3^). (For interpretation of the references to colour in this figure legend, the reader is referred to the web version of this article.)

In the northern sites (Beijing and Shanxi), air pollution exposures were similar in the heating season but higher in Shanxi in the non-heating season (Fig. 1). Guangxi participants had the lowest exposures to PM_2.5_, however, their exposures to black carbon were similar to or higher than northern participants in the same season (Supplementary Table 1). Air pollution exposures were higher among men (Table 2), though this gender difference was largely eliminated after accounting for active tobacco smoking (Supplementary Fig. S6). Participants exclusively using clean fuel for cooking, heating, or all energy use had exposures that were similar to users of solid fuel (Table 2).

**Table 2:** Geometric mean [and 95% confidence intervals] personal exposures to PM_2.5_ and black carbon (μg/m^3^) in *peri*-urban Chinese adults by season, gender, and household fuel use.

	**Heating season**^a^			**Non-heating season**^a^		
**Exposure group**	**N_participants_ (N_filters)_**	**PM_2.5_**	**Black carbon**	**N_participants_ (N_filters)_**	**PM_2.5_**	**Black carbon**
All participants	443 (785)	108 [100,116]	1.7 [1.6,1.8]	738 (1291)	65 [62,68]	1.1 [1.0,1.1]
Men	201 (340)	122 [110,135]	1.7 [1.5,2.0]	320 (566)	72 [67,77]	1.1 [1.0,1.2]
Women	241 (444)	98 [88,108]	1.6 [1.5,1.8]	412 (723)	61 [58,64]	1.0 [1.0,1.1]
Exclusive use of clean fuel^b^ for cooking	238 (431)	101 [91,113]	1.8 [1.6,2.0]	336 (584)	64 [60,69]	1.0 [0.9,1.1]
Use of solid fuel^b^ for cooking	197 (344)	113 [103,124]	1.6 [1.4,1.7]	376 (672)	67 [63,71]	1.2 [1.1,1.3]
Exclusive use of clean fuel for heating	116 (208)	108 [95,122]	1.7 [1.5,2.0]	205 (363)	67 [62,73]	1.2 [1.1,1.4]
Use of solid fuel for heating, indoor stoves	271 (482)	109 [100,119]	1.7 [1.5,1.9]	301 (522)	71 [65,76]	0.9 [0.8,1.0]
Use of solid fuel for heating, outdoor stoves	35 (60)	86 [54,137]	1.4 [1.0,1.9]	33 (55)	71 [57,88]	0.8 [0.6,1.1]
Exclusive use of clean fuel for cooking and heating	73 (134)	114 [97,133]	1.9 [1.6,2.2]	165 (286)	66 [61,72]	1.3 [1.2,1.4]
Use of solid fuel for cooking and/or heating	362 (641)	105 [97,114]	1.6 [1.5,1.8]	547 (970)	66 [62,69]	1.0 [0.9,1.1]

PM, particulate matter.

a Heating season includes measurements from northern sites only; non-heating season includes measurements from all 3 sites. The 2 24-h measurements were averaged to estimate ‘daily’ within-season exposure for each participant. We used the single 24-h measurement if 2 complete measurements were not available.

b Clean fuel refers to gas and/or electricity and solid fuel refers to use of biomass and/or coal.

### 3.3 Outdoor PM_2.5_

Daily outdoor PM_2.5_ (from government monitors) ranged from 6 to 407 μg/m^3^ (geometric mean (GM): 67) (Supplementary Table S2, Supplementary Fig. S7). In the heating season, Beijing and Shanxi had similar outdoor PM_2.5_ (GM: 55 and 54 μg/m^3^, respectively). In the non-heating season, Shanxi had the lowest outdoor PM_2.5_ (GM: 22 μg/m^3^ compared with 38 μg/m^3^ in Guangxi and 45 μg/m^3^ in Beijing). Average (GM) personal exposures were consistently higher than average outdoor PM_2.5_ in the same season (+38 μg/m^3^ in Beijing heating season; +49 μg/m^3^ in Shanxi heating season; +17 μg/m^3^ higher in Guangxi non-heating season; +17 μg/m^3^ in Beijing non-heating season; and + 59 μg/m^3^ in Shanxi non-heating season).

### 3.4 Variance components of personal exposure to PM_2.5_ and black carbon

In the base intercept-only models, the proportion of total variability in air pollution exposure attributed to between-individual differences was low to moderate (range of ICCs: 0.05–0.32), with consistently greater within-individual variability than between-individual variability (Table 3). Compared with models including all observations, the ICCs were similar for gender-specific models (range: 0.05–0.14) but higher in season-specific models (0.29–031), indicating that day-to-day measurements within the same season are more similar than measurements for the same individual in different seasons. The ranges of ICCs were similar for models predicting PM_2.5_ (0.08–0.29) versus black carbon (0.07–0.32).

**Table 3:** Estimates of between-individual and within-individual components of variance of 24-h measurements of personal exposure to PM_2.5_ and black carbon from random intercept-only models.

	Models predicting PM_2.5_					Models predicting black carbon				
	All obs	Women	Men	Heating	Non-heating	All obs	Women	Men	Heating	Non-heating
Mean (ln(µg/m^3^); 95% CI	4.3 4.2–4.3	4.2 4.1–4.2	4.4 4.3–4.4	4.6 4.5–4.6	4.1 4.1–4.1	0.1 0.1–0.2	0.1 0.0–0.1	0.2 0.1–0.2	0.4 0.3–0.5	−0.1 −0.1–0.0
Between-individual variance (σ_b_^2^)	0.10	0.07	0.12	0.35	0.21	0.10	0.12	0.07	0.35	0.33
Within-individual variance (σ_ε_^2^)	0.80	0.83	0.77	0.76	0.48	1.10	1.04	1.19	0.85	0.80
ICC	0.11	0.07	0.14	0.32	0.31	0.08	0.10	0.05	0.29	0.29

CI, confidence interval; ICC, intraclass correlation coefficient; obs, observations; PM, particulate matter.

Notes: The ICC is the proportion of total variability in exposure attributed to between-individual differences.

### 3.5 Model fit and performance

The within-individual variance remained much larger than the between-individual variance, even after including outdoor air quality and other time-varying variables in the models (*σ_ε_*^2^ = 0.65–1.11; *σ_b_*^2^ = 0.05–0.13) (Table 4). Outdoor PM_2.5_ explained the largest proportion of within-individual variance relative to the PM_2.5_ intercept-only model (+16%). The addition of other time-varying variables including season, outdoor temperature, and relative humidity had limited additional explanatory power (+2% for PM_2.5_ and + 5% for black carbon). Indoor sources (smoking status and household fuel type) and study site explained the largest proportion of between-individual variability in PM_2.5_, while outdoor PM_2.5_ had little impact. Adding indoor sources and other time-invariant variables into the black carbon models had little impact on the explained between-individual variance. Socio-demographic variables including age, gender, occupation, marital status, education, and income had little to no explanatory power. Compared with the intercept-only models, the full models explained an additional 20% and 5% of within-individual variance and an additional 46% and 11% of between-individual variance in exposure to PM_2.5_ and black carbon, respectively.

**Table 4:** Model prediction and fit of linear mixed effect models predicting personal exposure to PM_2.5_ and black carbon (BC) in *peri*-urban Chinese adults.

		Prediction	Fit					
		Within-individual variance (*σ_e_*^2^)	Between-individual variance (*σ_b_*^2^)	R^2^_within_^a^	R^2^_between_^b^	ICC (ρ)*	RMSE	Spearman correlation
Base random intercept model $\ln\left(Y_{\mathrm{ik}}\right)=\beta_{0}+b_{i}+\varepsilon_{\mathrm{ik}}$	PM_2.5_	0.81	0.10	Ref	Ref	0.11	0.86	0.60
	BC	1.11	0.09	Ref	Ref	0.07	1.02	0.55
Base + outdoor PM_2.5_ $\ln\left(Y_{\mathrm{ik}}\right)=\beta_{0}+{{\beta_{1}\left(outdoor\right)}_{i}+b}_{i}+\varepsilon_{\mathrm{ik}}$	PM_2.5_	0.68	0.13	0.16	−0.32	0.16	0.78	0.66
	BC	0.95	0.10	0.00	0.00	0.10	0.94	0.62
Base + outdoor PM_2.5_ + temp. + RH $\ln\left(Y_{\mathrm{ik}}\right)=\beta_{0}+{{\beta_{1}\left(outdoor\right)}_{i}+{\beta_{2}\left(temp.\right)}_{i}+{\beta_{3}\left(RH\right)}_{i}+b}_{i}+\varepsilon_{\mathrm{ik}}$	PM_2.5_	0.68	0.13	0.17	−0.36	0.16	0.77	0.67
	BC	0.94	0.12	0.01	−0.11	0.11	0.93	0.63
Base + outdoor PM_2.5_ + temp. + RH + season $\ln\left(Y_{\mathrm{ik}}\right)=\beta_{0}+{{\beta_{1}\left(outdoor\right)}_{i}+{\beta_{2}\left(temp.\right)}_{i}+{\beta_{3}\left(RH\right)}_{i}+{\beta_{2}\left(season\right)}_{\mathrm{ik}}+b}_{i}+\varepsilon_{\mathrm{ik}}$	PM_2.5_	0.66	0.10	0.18	−0.01	0.13	0.77	0.65
	BC	0.91	0.11	0.05	−0.06	0.11	0.91	0.64
Base + outdoor PM_2.5_ + temp. + RH + season + fuel^c^ $ln\left(Y_{\mathrm{ik}}\right)=\beta_{0}+{\beta_{1}\left(outdoor\right)}_{i}+{\beta_{2}\left(temp.\right)}_{i}+{\beta_{3}\left(RH\right)}_{i}+{\beta_{4}\left(season\right)}_{i}+{\beta_{5}\left(fuel\right)}_{\mathrm{ik}}+b_{i}+\varepsilon_{\mathrm{ik}}$	PM_2.5_	0.66	0.09	0.19	0.07	0.12	0.77	0.65
	BC	0.91	0.10	0.05	0.05	0.10	0.91	0.61
Base + outdoor PM_2.5_ + temp. + RH + season + fuel^c^ + smoke $ln\left(Y_{\mathrm{ik}}\right)=\beta_{0}+{\beta_{1}\left(outdoor\right)}_{i}+{\beta_{2}\left(temp.\right)}_{i}+{\beta_{3}\left(RH\right)}_{i}+{\beta_{4}\left(season\right)}_{i}+{\beta_{5}\left(fuel\right)}_{\mathrm{ik}}+{\beta_{6}\left(smoke\right)}_{\mathrm{ik}}+b_{i}+\varepsilon_{\mathrm{ik}}$	PM_2.5_	0.66	0.06	0.19	0.35	0.09	0.78	0.64
	BC	0.91	0.09	0.05	0.10	0.09	0.92	0.61
Base + outdoor PM_2.5_ + temp. + RH + season + fuel^c^ + smoke + site $lln\left(Y_{\mathrm{ik}}\right)=\beta_{0}+{\beta_{1}\left(outdoor\right)}_{i}+{\beta_{2}\left(temp.\right)}_{i}+{\beta_{3}\left(RH\right)}_{i}+{\beta_{4}\left(season\right)}_{i}+{\beta_{5}\left(fuel\right)}_{\mathrm{ik}}+{\beta_{6}\left(smoke\right)}_{\mathrm{ik}}+{\beta_{7}\left(site\right)}_{\mathrm{ik}}+b_{i}+\varepsilon_{\mathrm{ik}}$	PM_2.5_	0.65	0.05	0.20	0.46	0.07	0.78	0.66
	BC	0.91	0.09	0.05	0.11	0.09	0.92	0.60
Full model: Base + outdoor PM_2.5_ + temp. + RH + season + fuel^c^ + smoke + site + all other covariates^d^ $ln\left(Y_{\mathrm{ik}}\right)=\beta_{0}+{\beta_{1}\left(outdoor\right)}_{i}+{\beta_{2}\left(temp.\right)}_{i}+{\beta_{3}\left(RH\right)}_{i}+{\beta_{4}\left(season\right)}_{i}+{\beta_{5}\left(fuel\right)}_{\mathrm{ik}}+{\beta_{6}\left(smoke\right)}_{\mathrm{ik}}+{\beta_{7}\left(site\right)}_{\mathrm{ik}}+{\beta_{8}\left(X\right)}_{\mathrm{ik}}+b_{i}+\varepsilon_{\mathrm{ik}}$	PM_2.5_	0.65	0.05	0.20	0.46	0.07	0.77	0.66
	BC	0.91	0.09	0.04	0.11	0.09	0.92	0.60

PM, particulate matter; BC, black carbon; ICC, intraclass correlation; R^2^, coefficient of determination; temp, temperature; RH, relative humidity.

a Within-individual variance explained relative to the intercept-only model.

b Between-individual variance explained relative to the intercept-only model.

c Variables for cooking and heating fuel were added separately into the models.

d Includes participant age, gender, occupation, marital status, education, and income.

The RMSE between the natural logged-transformed predicted and measured air pollution exposures decreased as covariates were successively added into the models (from 0.86 to 0.77 for PM_2.5_ and from 1.02 to 0.92 for black carbon when comparing the base and full models, respectively), indicating small increases in predictive validity (Table 4). We also observed small increases in the Spearman correlation (from 0.60 to 0.66 for PM_2.5_ and from 0.55 to 0.60 for black carbon).

We continued to observe strong seasonal and regional patterns in pollution exposures in the multivariable models (Table 5). Exposures to PM_2.5_ and black carbon in the non-heating season were 63% lower (95% CI: −72%, −51%) and 78% lower (95% CI: −84%, −69%) than the heating season, respectively, even after accounting for outdoor air quality, temperature, and humidity. Participants in Beijing and Shanxi had 38% and 70% higher exposure to PM_2.5_ than participants in Guangxi, respectively, though the opposite trend was observed for black carbon (compared with Guangxi, black carbon exposures were 13% and 18% lower in Beijing and Shanxi, respectively).

**Table 5:** Associations between personal exposures to air pollution and selected sociodemographic, energy use, and environmental variables.^a^

	Percent (%) change in PM_2.5_ based on log regression* (95% CI) (n = 2022 filters)	Percent (%) change in black carbon based on log regression* (95% CI) (n = 2026 filters)
**Age, per year**	−0.2 [−0.8,0.4]	0.0 [−0.7, 0.8]
**Gender**		
male (ref: female)	4.6 [−6.1, 16.5]	6.5 [−6.5, 21.4]
**Occupation**		
agriculture (ref)		
other work outside the home	5.9 [−10.3, 24.9]	14.1 [−6.6, 39.3]
not working outside the home	−3.2 [−13.1, 7.8]	−6.1 [−17.6, 7.0]
**Annual household income**		
(yuan)		
<20000 (ref: ≥20000)	3.1 [−5.9, 13.0]	1.6 [−9.1, 13.6]
**Education**		
college/high (ref)		
primary	0.4 [−8.7, 10.5]	4.5 [−6.9, 17.3]
no school	−5.4 [−17.2, 8.1]	7.2 [−8.8, 26.1]
**Smoking status**		
smoker (ref)		
non-smoker w/ household smoker	−26.2 [−36.3, −14.4]***	−1.3 [−17.5, 8.2]
non-smoker w/o household smoker	−30.4 [−38.0, −21.8]***	−12.8 [−24.2, 0.3]*
**Cooking fuel**		
clean fuel use (ref: any solid fuel)	−15.4 [–22.3, −8.0]***	−14.8 [–23.1, −5.6]***
**Heating fuel**		
indoor solid fuel (ref)		
outdoor solid fuel use	−24.6 [−36.98, −9.8]***	−19.9 [−35.0, −0.5]**
only clean fuel	−2.8 [−12.3, 7.7]	5.6 [−6.7, 19.6]
no devices	−1.6 [−16.6, 16.3]	2.1 [−16.5, 24.8]
**Season**		
non-heating (ref: heating)	−62.8 [−71.8, −50.9]***	−78.0 [−84.2, −69.3]***
**Outdoor PM_2.5,_ per 10** μ**g/m^3^**	5.8 [4.7, 6.9]***	7.9 [6.6, 9.2]***
**Ambient RH, per 1%**	0.8 [0.5, 1.1]***	0.0 [−0.3, 0.4]
**Ambient temperature, per 1 °C**	3.4 [2.2, 4.6]***	5.5 [4.1, 7.0]***
**Site**		
Guangxi (ref)	37.9 [15.0, 65.3]***	
Beijing		−12.8 [30.0, 8.5]
Shanxi	69.9 [43.1, 101.6]***	−18.4 [–33.7, 0.3]*
**Marginal R^2^**	0.24	0.17
**Conditional R^2^**	0.29	0.24

*p-value < 0.10; **p-value < 0.05; ***p-value < 0.001; obs, observations.

a Regression of log-air pollution exposure can be converted to the percent (%) change in exposure using the equation ([exp^β^ – 1] x 100), where β is the change in log-transformed pollution exposure associated with a one-unit change in the independent variable.

Indoor sources including household fuel type and smoking patterns were strongly associated with exposures. Participants exclusively cooking with gas and electric stoves had 15% lower exposure to PM_2.5_ and black carbon than users of solid fuel stoves. Compared with participants using solid fuel heating stoves indoors, participants with outdoor stoves had 25% lower (95% CI: −37%, −10%) exposure to PM_2.5_ and 20% lower (95% CI: −35%, −0.5%) exposure to black carbon, though no differences were observed for users of clean fuel heating stoves or without heating-specific stoves. Poor outdoor air quality was associated with higher exposure [6% higher PM_2.5_ (95% CI: 5%, 7%) and 8% higher black carbon (95% CI: 7%, 9%) per 10 μg/m^3^ increase in outdoor PM_2.5_]. Participants that were male, had lower household incomes, or that worked outside of the home had 2–14% higher exposures to air pollution, though the differences were not statistically significant.

The gender-specific models were very similar to the full models with the exception of outdoor solid fuel heating stove use which, compared with use of indoor solid fuel heating stoves, was associated with lower exposures in women (-37%; 95% CI: −51%, −20%) but not in men (Supplementary Table S3). Season-specific models suggest that outdoor air quality may have a larger impact on exposure in the non-heating season than the heating season (10% versus 4% higher exposure per 10 μg/m^3^ increase in outdoor PM_2.5_). We did not observe any qualitative differences in our results after excluding observations that did not capture ± 10% of the target 24-h sampling time, or when comparing results from models with measurement versus estimated outdoor PM_2.5_ (Supplementary Table S4).

## 4 Discussion

We conducted one of the largest and most comprehensive household air pollution exposure studies to date, which included over 2073 measurements of 24-h personal exposures to PM_2.5_ and black carbon from 778 participants. By conducting repeated measurements across seasons, we were able to describe the levels and variability in PM_2.5_ exposures in *peri*-urban men and women living in 3 diverse provinces of China and also assess the explanatory contribution of indoor and outdoor sources to variability and levels of personal exposures.

Personal exposures to PM_2.5_ in our study were within the range of exposures observed among non-smoking women cooking with biomass stoves in southwestern China (range of GMs: 47–91 μg/m^3^ in summer and 107–201 μg/m^3^ in winter)(Baumgartner et al., 2019, Baumgartner et al., 2014, Ni et al., 2016) but were higher than exposures among urban Chinese (range of means: 33–93 μg/m^3^)(Lin et al., 2020, Lei et al., 2020, Chen et al., 2020). Outdoor PM_2.5_ was high in our study settings, exceeding the WHO’s 24-h Air Quality Guideline on 56% of study measurement days. Though our finding that personal exposures were consistently higher than outdoor air pollution, particularly in northern China, highlights the contribution of indoor sources to exposures in settings with poor outdoor air quality.

Large and consistent differences in outdoor air quality and exposures were observed by geographic region. Of our 3 study sites, Guangxi participants (southern China) had the lowest exposures to PM_2.5_ in the non-heating season but the highest exposures to black carbon. This result may be in part due to the higher proportion of Guangxi participants exclusively using clean fuel stoves (31% versus 19% in the northern sites) and their more common use of biomass stoves which can emit proportionally higher levels of black carbon compared with coal stoves (Zhang et al., 2018, Shen et al., 2010). Guangxi participants also lived in homes that were closer to major roadways (3–11 km) and secondary roads, which may also have influenced their exposures to black carbon (Google. Google Maps Guangxi, China, 2020, Zheng et al., 2017). Planned chemical analysis of a sub-sample of PTFE filters from the study will provide a better understanding of the source-specific contributors to exposures in our study.

In northern China, air pollution exposures were twice as high in the heating season than the non-heating season, a result that is likely in part attributable to space heating stove emissions and potentially due to less time spent outside the home. The role of these very high seasonal exposures on health, particularly for cardiovascular diseases, should be further investigated. Seasonal variability of cardiovascular diseases is well-documented in China and elsewhere, showing mostly a peak in winter months (Fares, 2013, Stewart et al., 2017). The exact causes of these seasonal differences are not fully understood, though environmental factors like air pollution are strongly associated with cardiovascular outcomes and thus may play some role (Fares, 2013). Replacing traditional coal and biomass space heating stoves with electric or gas appliances may therefore benefit both outdoor and indoor air quality and population health in northern China (Barrington-Leigh et al., 2019).

Both active smoking and environmental tobacco smoke were important contributors to exposure, with the former impacting men and the latter impacting women. Men in our study had higher exposures than women, on average, though exposures among non-smoking women and men were similar. Policy organizations including the WHO consistently highlight the high levels of household air pollution exposures among women and children (World Health Organization, 2018), but very few studies have measured exposure in men (Shupler et al., 2018, Kaptoge et al., 2019, Balakrishnan et al., 2019). The gender-specific results from our study align with measurements of PM_2.5_ exposure in largely non-smoking men in *peri*-urban India, which were similar to women (55 versus 58 µg/m^3^). By comparison, women in a small exposure study conducted in rural Ethiopia and Uganda had exposures to PM_2.5_ that were 5–6 times higher than men in the same villages. Women in our study sites were usually the primary cooks, though men can be in close proximity to cooking stoves even if they are not cooking themselves. Men also participated in other household energy tasks. For example, men were often responsible for operating space heating stoves where they can be episodically exposed to high levels of pollution during fuel loading. This practice is reflected in our gender-specific models, where use of an outdoor solid fuel heating stove (compared with an indoor solid fuel stove) was associated with proportionally lower exposure in women (-37%; 95% CI: −51, −20) than in men (-10%; 95% CI:-31, −18), likely because men were still highly exposed to outdoor stove emissions during re-fueling.

Participants living in homes without smokers had considerably lower exposures than smokers (30% lower for PM_2.5_ and 13% lower black carbon). The proportionally larger difference for PM_2.5_ may be due to the large organic fraction in tobacco smoke (Schauer, 2003) which contributes to higher PM_2.5_ but not higher black carbon. A somewhat surprising finding in the crude (unadjusted) analysis was that participants in homes exclusively cooking or heating with clean fuel stoves still had high exposures to PM_2.5_ (GM: 76 µg/m^3^; range: 11 – 392 µg/m^3^) that were similar to participants using solid fuel stoves (GM: 81 µg/m^3^; range: 3 – 838 µg/m^3^). After statistically accounting for outdoor air quality and other variables, exclusive users of clean fuel cookstoves and heating stoves had only modestly lower exposures than indoor solid fuel users (-3 to −15% for PM_2.5_). These results provide further empirical evidence that poor outdoor air quality and other behavioral factors can mask the benefit of clean energy use, but also highlight the importance of evaluating all major sources of air pollution in intervention studies to better understand their relative contributions to exposures and also have more realistic expectations of the air pollution exposure benefits of a clean stove interventions in settings where other sources are also present.

We observed high within-individual variability in 24-h exposure across seasons and within the same season, particularly when compared with between-individual variance. Based on our mixed-effects models, we partly attribute this finding to the high day-to-day variability in outdoor air quality, though a large portion of within-individual variance remained unexplained in the full models. The ICCs in our base and covariate-adjusted models (PM_2.5_: 0.11–0.16; black carbon: 0.08–0.11) were lower than those observed for carbon monoxide exposure among children in The Gambia (ICC = 0.33) (Dionisio et al., 2012), but overlapped with those among adults in Guatemala (carbon monoxide ICC: 0.11–0.33) (McCracken et al., 2009) and *peri*-urban India (PM_2.5_ ICC: 0.0–0.22) (Sanchez et al., 2019). As expected, the ICCs in our study were higher in the season-specific models, but still indicated poor reliability (range: 0.29–0.32). Overall, our results indicate that a single day of measured exposure is not likely representative of longer-term exposure, which is the exposure metric most relevant for many chronic health outcomes including cardiovascular diseases (Health Effects Institute, 2019, Jaganathan et al., 2019). They also highlight the challenges of identifying the impact of any given source on personal exposure in these complex air pollution settings where behaviors like time spent in different locations or doing certain activities are likely important determinants of exposure that are not easily captured by traditional survey methods and measurements (Milà et al., 2018).

Notable strengths of this study include the comprehensive dataset of over 48,000 h of personal exposure monitoring in 3 diverse provinces of China which includes measurements of exposure among men and exclusive clean fuel users in villages using solid fuel energy. Despite the considerable practical and logistical challenges of conducting large panel studies of exposure in these settings (Clark et al., 2013), we were able to obtain at least 2 days of measured air pollution exposures for 90% of participants and 4 days for 60% of northern China participants, which allowed us to evaluate the within-individual and between-individual variance in daily exposure. These results contribute to the very limited evidence on representativeness of short-term measurement of exposure for longer-term exposure estimation in field studies of household air pollution. Further, the additional assessment of very detailed energy use and outdoor PM_2.5_ allowed us to evaluate the influence of indoor versus outdoor sources to personal exposures, which are contributions to exposures that remain poorly understood in many settings, especially relative to one another.

Our study is not without limitations. Though we achieved high compliance in wearing the air monitors (98% in participants randomly selected for compliance monitoring with a pedometer), it is possible that some participants altered their daily activity patterns while wearing them. We also cannot rule out the possibility that wearing the monitors or visiting the clinics may have changed participants’ behaviors. We were unable to account for time-varying behaviors which are likely important determinants of exposure in our study participants such as stove use on the measurement days or time-activity patterns. Combined use of GPS monitors or Bluetooth signal receivers can track participant location during measurement and allow investigators to better assess activity patterns in field studies(Liao et al., 2019), though the required data processing and analysis can be time-intensive in large studies like this one. We were limited to 2 days of measurement per season due to study logistics and participant burden in wearing the monitors, which limited our ability to assess ‘long-term’ exposure over weeks or months. The recent development of quiet and less bulky PM_2.5_ monitors may ease some of the logistical and participant burdens of longer-term measurements. In addition to the detailed fuel and stove use data collection in this study, future studies could also collect information on home ventilation which was not collected in this study.

## 5 Conclusion

Personal exposures to PM_2.5_ across all seasons and study sites were, on average, higher than the WHO’s 24-h PM_2.5_ air quality guideline and higher than the relatively high levels of outdoor PM_2.5_. Our repeated measures show that within-individual variance dominated the total variability in personal exposures across all study sites, genders, and seasons. Repeated daily measurements of exposure are thus needed to capture ‘usual’ daily exposure for epidemiological and intervention studies in these settings, even within a single season. Our results also indicate that measurably reducing air pollution exposures in these study settings will likely require reductions in emissions from both indoor and outdoor air pollution, which are linked to different air pollution mitigation policies and interventions.

## CRediT authorship contribution statement

**Martha Lee:** Data curation, Formal analysis, Writing - original draft, Writing - review & editing. **Ellison Carter:** Resources, Data curation, Writing - review & editing. **Li Yan:** Data curation, Writing - review & editing, Project administration. **Queenie Chan:** Writing - review & editing, Project administration. **Paul Elliott:** Funding acquisition, Writing - review & editing. **Majid Ezzati:** Funding acquisition, Writing - review & editing. **Frank Kelly:** Funding acquisition, Writing - review & editing. **James J. Schauer:** Resources, Data curation, Writing - review & editing. **Yangfeng Wu:** Resources, Funding acquisition, Writing - review & editing. **Xudong Yang:** Resources, Funding acquisition, Writing - review & editing. **Liancheng Zhao:** Resources, Funding acquisition, Writing - review & editing. **Jill Baumgartner:** Conceptualization, Methodology, Writing - original draft, Supervision, Project administration, Funding acquisition, Writing - review & editing.

## Declaration of Competing Interest

The authors declare that they have no known competing financial interests or personal relationships that could have appeared to influence the work reported in this paper.
