# Supplementary material for: Determinants of personal exposure to PM2.5 and black carbon in Chinese adults: A repeated-measures study in villages using solid fuel energy
Source: Environ Int. 2021 Jan;146:106297. doi: 10.1016/j.envint.2020.106297 (PMC7762838; doi:10.1016/j.envint.2020.106297)
Supplement: Supplementary data 1 [file mmc1.docx]

**Supplemental Material**

**Figure S1.** Location of study sites in the INTERMAP China Prospective (ICP) Study

**Figure S2:** Setup of the Harvard Personal Exposure Monitor within waistpacks (Photo credit: Ellison Carter)

**Figure S3.** Scatterplot of outdoor 24-h average PM_2.5_ estimated from government monitors using inverse distance weighting versus measured outdoor PM_2.5_ collected in the villages

**Figure S4.** Scatterplot of outdoor 24-h average temperature estimated from government monitors using inverse distance weighting versus measured outdoor temperatures taken during the clinic visits

**Figure S5.** Correlations between (A) exposures to PM_2.5_ and black carbon (BC) on the same day for all participants and between (B) PM_2.5_ and (C) black carbon exposures on subsequent sampling days for the same participants

**Figure S6:** Personal exposure to PM_2.5_ by study site and gender in the heating (A) and non-heating (B) seasons

**Figure S7.** Distributions of average 24-h outdoor PM_2.5_ (estimated from government monitors) in the heating and non-heating seasons at our study sites

**Table S1:** Personal exposures to PM_2.5_ and black carbon (BC) in peri-urban Chinese adults by socio-demographic and energy use variables (geometric means [95% CI]) in μg/m^3^)

**Table S2.** Comparison of outdoor PM_2.5_ (μg/m^3^) measured by village monitors and estimated from data provided by nearly government monitoring stations, averaged across study sites

**Table S3.** Associations between personal PM_2.5_ exposure and selected sociodemographic, energy use, and environmental variables by gender and season, expressed as % change in exposure [95% CI]

**Table S4.** Sensitivity analysis of associations between personal PM_2.5_ exposure and selected sociodemographic, energy use, and environmental variables, expressed as % change in exposure [95% CI]

**
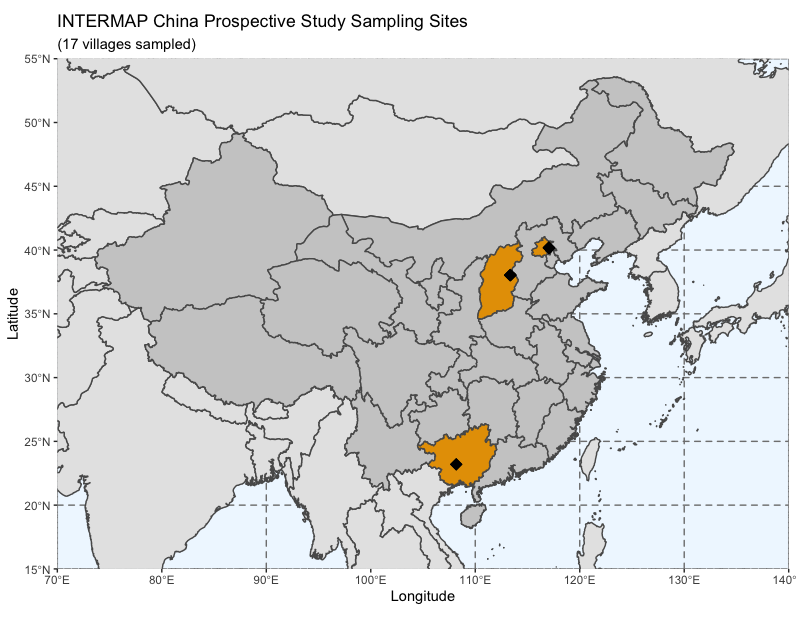
**

**Guangxi**

**(villages = 3)**

**Beijing**

**(villages = 8)**

**Shanxi**

**(villages = 6)**

**Figure S1. Location of study sites in the INTERMAP China Prospective (ICP) Study**


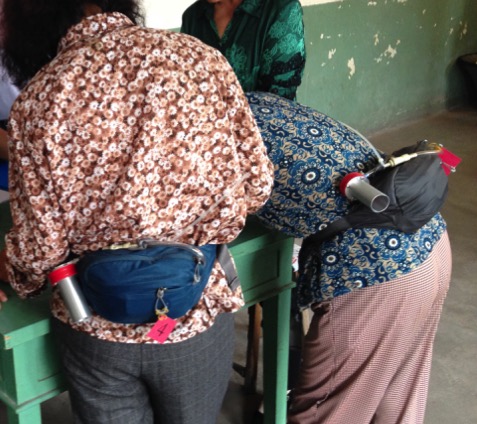


**Figure S2.** Setup of the Harvard Personal Exposure Monitor within waistpacks (Photo credit: Ellison Carter)

**Figure S3. Scatterplot of outdoor 24-h average PM_2.5_ estimated from government monitors using inverse distance weighting versus measured outdoor PM_2.5_ collected in the villages**

**Figure S4. Scatterplot of outdoor 24-h average temperature estimated from government monitors using inverse distance weighting versus measured outdoor temperatures taken during the clinic visits**


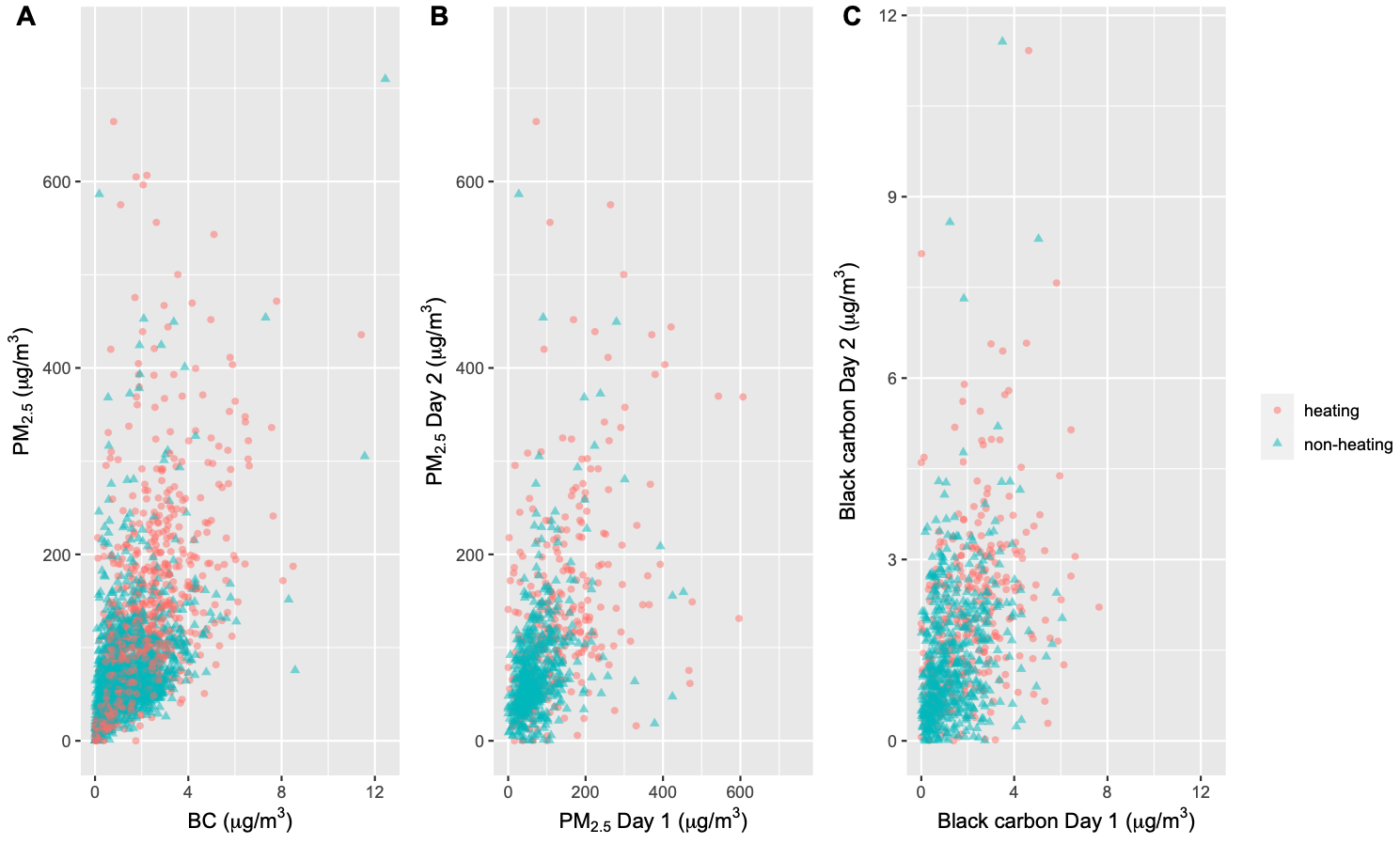


r = 0.49

r = 0.40

r = 0.44

**Figure S5. Correlations between (A) exposures to PM_2.5_ and black carbon (BC) on the same day for all participants and between (B) PM_2.5_ and (C) black carbon exposures on subsequent sampling days for the same participants**

| A Heating season   | B Non-heating season   |
| --- | --- |
| **Figure S6: Personal exposure to PM_2.5_ by study site and gender in the heating (A) and non-heating (B) seasons**  Personal exposures consisted of at least one observation per participant, but if the participant had two measurements from that season, they were both averaged. The figure’s y-axis was limited at 605 μg/m^3^ which remove 1 observation from the heating season (838 μg/m^3^ – a male non-smoker with no household smokers from Shanxi) and 2 observations in the non-heating season (710 μg/m^3^ – a male non-smoker with no household smokers from Beijing and 1241 μg/m^3^ – a male smoker from Shanxi). | |

Red line: World Health Organization 24-h guideline for outdoor PM_2.5_ (25 μg/m^3^)

**Figure S7. Distributions of average 24-h outdoor PM_2.5_ (estimated from government monitors) in the heating and non-heating seasons for all study sites**

|  | **Guangxi** | | **Beijing** | | | | **Shanxi** | | | |
| --- | --- | --- | --- | --- | --- | --- | --- | --- | --- | --- |
|  | **Non-heating** | | **Non-heating** | | **Heating** | | **Non-heating** | | **Heating** | |
|  | **PM_2.5_** | **BC** | **PM_2.5_** | **BC** | **PM_2.5_** | **BC** | **PM_2.5_** | **BC** | **PM_2.5_** | **BC** |
| **# of participants (# of filters)** | 238 (442) | 238 (441) | 228 (383) | 228 (384) | 237 (432) | 237 (433) | 269 (467) | 270 (468) | 205 (350) | 206 (352) |
| **All samples** | 51[48, 55] | 1.2 [1.1,1.3] | 59.4 [54,65] | 1.0 [0.9,1.1] | 103 [94,112] | 1.8 [1.6,2.0] | 88 [82,94] | 1.0 [0.9,1.1] | 114 [101,128] | 1.6 [1.4,1.74] |
| **Age**  40 – 49  50 – 59  60 – 69  70 – 79 | 59 [50,69]  51 [44,60]  51 [46,57]  49 [42,56] | 1.2 [0.9,1.5]  1.1 [0.9,1.5]  1.2 [1.1,1.4]  1.3 [1.0,1.6] | 54 [31,95]  71 [59,84]  55 [49,63]  57 [47,68] | 0.7 [0.2,2.8]  1.1 [0.9,1.4]  0.8 [0.7,1.0]  1.0 [0.8,1.2] | 70 [53,92]  72 [61,86]  122 [107,139]  128 [108,153] | 1.9 [1.3,2.6]  1.3 [1.1,1.6]  1.9 [1.6,2.3]  2.3 [1.9,2.8] | 79 [66,94]  97 [84,111]  88 [80,97]  81 [72,90] | 0.7 [0.4,1.0]  1.0 [0.9,1.3]  1.1 [1.0,1.3]  1.1 [0.9,1.3] | 66 [32,138]  129 [110,151]  118 [101,137]  118 [92,151] | 1.4 [0.9,2.4]  1.8 [1.6,2.2]  1.5 [1.3,1.8]  1.4 [1.1,1.8] |
| **Gender**  male  female | 56 [50,62]  48 [44,52] | 1.4 [1.2,1.6]  1.1 [1.0,1.3] | 67 [58,77]  55 [49,61] | 1.0 [0.8,1.2]  0.9 [0.8,1.1] | 110 [95,128]  98 [87,110] | 1.7 [1.4,2.1]  1.9 [1.7,2.1] | 95 [85,107]  82 [77,88] | 1.0 [0.8,1.2]  1.1 [1.0,1.2] | 135 [117,157]  97 [82,116] | 1.8 [1.6,2.1]  1.4 [1.2,1.6] |
| **Ethnicity**  Han  Zhuang  other | 49 [44,54]  54 [49,60]  - | 1.2 [1.0,1.4]  1.3 [1.1,1.5]  - | 60 [55,65]  -  33 [14,78] | 1.0 [0.9,1.1]  -  0.5 [0.0,23.5] | 103 [94,113]  -  105 [100,109] | 1.8 [1.6,2.0]  -  1.6 [0.0,387] | 88 [82,94]  -  - | 1.0 [0.9,1.1]  -  - | 114 [101,128]  -  - | 1.6 [1.4,1.7]  -  - |
| **Current occupation**  agriculture  other work outside the  home  not working outside the  home | 55 [50,61]  58 [46,73]  49 [46,54] | 1.4 [1.2,1.7]  1.2 [0.8,1.7]  1.2 [1.0,1.3] | 60 [54,66]  70 [48,102]  55 [42,72] | 1.0 [0.9,1.1]  0.9 [0.5,1.8]  0.9 [0.7,1.2] | 100 [90,111]  110 [82,147]  122 [99,152] | 1.7 [1.5,2.0]  1.9 [1.2,3.1]  2.3 [1.8,3.0] | 87 [82,94]  133 [90,198]  79 [68,91] | 1.1 [0.9,1.2]  1.6 [1.1,2.5]  0.8 [0.6,1.1] | 118 [102,136]  122 [76,193]  99 [79,122] | 1.6 [1.4,1.8]  1.9 [1.3,2.8]  1.4 [1.2,1.7] |
| **Household income**  <20000 RMB  ≥20000 RMB | 52 [48,56]  48 [41,57] | 1.2 [1.1,1.4]  1.1 [0.9,1.5] | 62 [54,71]  58 [51,65] | 1.1 [0.9,1.3]  0.9 [0.7,1.0] | 97 [85,110]  110 [96,124] | 1.8 [1.5,2.0]  1.8 [1.5,2.2] | 87 [78,97]  88 [82,95] | 0.9 [0.8,1.1]  1.1 [1.0,1.2] | 101 [83,122]  120 [104,140] | 1.3 [1.1,1.6]  1.7 [1.5,1.9] |
| **Highest education attained**  no formal  education  primary school  early high school  or college | 47 [38,59]  51 [46,56]  53 [48,59] | 1.1 [1.0,1.4]  1.2 [0.9,1.6]  1.3 [1.1,1.5] | 49 [40,59]  62 [53,72]  64 [56,74] | 0.9 [0.8,1.1]  0.9 [0.7,1.1]  1.0 [0.8,1.2] | 112 [95,131]  116 [97,138]  91 [79,104] | 1.7 [1.5,2.0]  2.1 [1.7,2.6]  1.8 [1.4,2.2] | 87 [70,107]  88 [80,96]  88 [80,97] | 0.9 [0.7,1.0]  1.3 [1.0,1.7]  1.1 [1.0,1.3] | 111 [82,148]  114 [98,132]  114 [91,142] | 1.7 [1.4,2.0]  1.6 [1.2,2.2]  1.5 [1.3,1.7] |
| **Smoke**  current smoker  not current w/  household smoker  not current w/o  household smoker | 68 [58,80]  49 [43,56]  48 [44,53] | 1.6 [1.3,2.0]  1.1 [1.0,1.4]  1.2 [1.0,1.3] | 74 [62,88]  57 [47,69]  56 [50,63] | 1.0 [0.8,1.3]  1.1 [0.9,1.3]  0.9 [0.8,1.0] | 119 [95,148]  104 [89,120]  95 [83,109] | 1.6 [1.1,2.2]  2.1 [1.8,2.4]  1.7 [1.5,2.0] | 118 [103,135]  83 [75,92]  77 [71,84] | 1.1 [0.9,1.4]  1.1 [0.9,1.3]  1.0 [0.8,1.1] | 155 [128,187]  100 [72,140]  99 [86,114] | 1.9 [1.5,2.3]  1.5 [1.1,1.9]  1.5 [1.3,1.7] |
| **Cooking fuel**  exclusive clean  solid fuel | 53 [47, 60]  51 [47, 56] | 1.3 [1.1, 1.5]  1.2 [1.1, 1.4] | 57 [51, 64]  63 [55, 73] | 0.9 [0.8, 1.1]  1.0 [0.9, 1.2] | 93 [82, 105]  119 [105, 135] | 1.7 [1.4, 1.9]  2.0 [1.7, 2.3] | 84 [76, 92]  91 [84, 100] | 0.9 [0.7, 1.0]  1.2 [1.0, 1.3] | 118 [95, 146]  109 [95, 124] | 1.9 [1.6, 2.2]  1.3 [1.2, 1.5] |
| **Heating fuel**  no device  exclusive clean  solid fuel (indoor)  solid fuel (only outdoor) | 53 [48, 58]  49 [44, 55]  -  - | 1.2 [1.1, 1.4]  1.3 [1.1, 1.5]  -  - | 82 [30,222]  69 [57, 83]  56 [50, 63]  58 [36, 94] | 1.2[0.1, 10.1]  1.2 [1.0, 1.4]  0.9 [0.8, 1.0]  1.1 [0.7, 1.6] | 70 [-,-]  100 [85, 118]  102 [90, 114]  108 [78, 150] | 0.6 [-,-]  1.8 [1.5, 2.1]  1.7 [1.5, 2.0]  2.3 [1.5, 3.8] | 94 [70, 125]  86 [78, 95]  90 [82, 98]  77 [39,152] | 1.6 [1.1, 2.4]  1.3 [1.1, 1.5]  0.9 [0.8, 1.1]  0.7 [0.4, 1.1] | 116 [74,184]  115 [95, 139]  121 [107, 137]  78 [60, 100] | 1.7 [1.2, 2.5]  1.6 [1.3, 2.0]  1.6 [1.4, 1.9]  1.1 [0.7, 1.7] |
| **Outdoor PM_2.5_**  0-49 μg/m^3^  50-99 μg/m^3^  100-149 μg/m^3^  > 150 μg/m^3^ | 44 [41,47]  56 [50,64]  -  - | 1.0 [0.8,1.1]  1.2 [1.0,1.4]  -  - | 36 [31,42]  76 [68,84]  96 [85,108]  132 [114,153] | 0.6 [0.5,0.7]  1.3 [1.1,1.5]  1.1 [0.8,1.5]  1.9 [1.1,3.5] | 63 [57,70]  147 [130,165]  159 [140,180]  214 [192,237] | 1.1 [0.9,1.2]  2.5 [2.3,2.8]  3.1 [2.7,3.5]  3.0 [2.2,4.1] | 83 [78,88]  113 [87,147]  -  - | 0.9 [0.9,1.0]  2.2 [1.7,2.8]  -  - | 78 [66,93]  -  147 [121,179]  125 [101,155] | 1.0 [0.9,1.2]  -  2.4 [2.1,2.7]  2.3 [2.1,2.7] |

**Table S1: Personal exposures to PM_2.5_ and black carbon (BC) in peri-urban Chinese adults by socio-demographic and energy use variables (geometric means [95% CI]) in μg/m^3^)**

|  |  | N_filters_ | Heating season | | Non-heating season | |
| --- | --- | --- | --- | --- | --- | --- |
| Outdoor |  |  |  |  |  |  |
| PM_2.5_ (estimated from  nearby monitors)^a^ | Mean [95% CI]  Geo Mean [95% CI]  Range | NA | 93 [62,124]  55 [39,76]  6 – 407 | | 42 [35,50]  33 [28,39]  8 – 151 | |
| PM_2.5_  (village subsample)^b^ | Mean [95% CI]  Geo Mean [95% CI]  Range | 33 | 76 [48,105]  47 [29,77]  2 – 267 | | 58 [42,73]  54 [39,75]  21 – 90 | |

**Table S2. Comparison of outdoor PM_2.5_ (μg/m^3^) measured by village monitors and estimated from data provided by nearly government monitoring stations, averaged across study sites**

Geo mean, geometric mean; PM=particulate matter

^a^ Estimated from daily outdoor PM_2.5_ measurements obtained from nearby government air monitoring stations. The heating season includes measurements from northern sites only, while the non-heating season includes measurements from all 3 sites

^b^ Filter-based measurements of village PM_2.5_ were collected during 1 season at each study site. In the heating season, village measurements were collected in Beijing and Shanxi. In the non-heating season, samples were collected in Guangxi.

| **N_participants_ (N_filters_)** | **All Participants**  **746 (2022)** | **Females**  **413 (1137)** | **Males**  **885 (333)** | **Heating season**  **771 (433)** | **Non-heating season**  **1251(709)** |
| --- | --- | --- | --- | --- | --- |
| **Age, per year** | -0.2 [-0.8,0.4] | -0.1 [-0.9, 0.7] | -0.4 [-1.3, 0.5] | 0.6 [-0.5, 1.8] | -0.4 [-1.1, 0.2] |
| **Gender**  male (ref: female) | 4.6 [-6.1, 16.5] | - | - | 16.2 [-5.0, 42.0] | -1.3 [-12.2, 10.9] |
| **Occupation**  agriculture (ref)  other work outside the home  not working outside the home | 5.9 [-10.3, 24.9]  -3.2 [-13.1, 7.8] | -12.3 [-35.6, 19.4]  -7.0 [-18.2, 5.9] | 11.7 [-9.4, 37.6]  0.3 [-18.4, 23.4] | 15.6 [-16.1, 59.2]  -4.0 [-21.0, 16.7] | -2.5 [-18.5, 16.6]  -4.0 [-14.9, 8.1] |
| **Annual household income**  **(yuan)**  <20000 (ref: ≥20000) | 3.1 [-5.9, 13.0] | 4.2 [-7.5, 17.3] | 0.0 [-13.9, 16.1] | 4.7 [-10.4, 22.2] | 6.0 [-4.6, 17.7] |
| **Education**  college/high (ref)  primary  no school | 0.4 [-8.7, 10.5]  -5.4 [-17.2, 8.1] | -5.4 [-17.7, 8.8]  7.6 [-21.7, 9.0] | 7.9 [-5.9, 23.7]  -6.3 [-29.0, 23.6] | -2.5 [-17.9, 15.8]  -6.9 [-25.7, 16.7] | 3.3 [-7.0, 14.8]  -3.5 [-17.2, 12.3] |
| **Smoking status**  smoker (ref)  non-smoker w/ household smoker  non-smoker w/o household smoker | -26.2 [-36.3, -14.4]***  -30.4 [-38.0, -21.8]*** | -24.7 [-49.2, 11.6]  -30.0 [-52.6, 3.2]* | -33.6 [-54.3, -3.4]**  -29.2 [-37.7, -19.6]*** | -16.3 [-36.1, 9.6]  -30.3 [-43.2, -14.5]*** | -30.5 [-41.0, -18.2]***  -29.0 [-37.6, -19.2]*** |
| **Cooking fuel**  clean fuel use (ref: solid fuel) | -15.4 [-22.3, -8.0]*** | -12.7 [-22.1, -2.1]** | -19.0 [-28.9, -7.8]*** | -17.4 [-29.2, -3.6]** | -11.3 [-19.2, -2.7]** |
| **Heating fuel**  indoor solid fuel (ref)  outdoor solid fuel use  only clean fuel  no devices | -24.6 [-36.9, -9.8]***  -2.8 [-12.3, 7.7]  -1.6 [-16.6, 16.3] | -37.3 [-51.1, -19.6]***  -8.1 [-19.5, 4.9]  0.6 [-19.0, 24.9] | -9.9 [-31.0, 17.7]  5.0 [-11.1, 24.0]  3.7 [-26.1, 25.3] | -37.8 [-52.7, -18.0]***  -8.1 [-22.1, 8.4]  0.7 [-33.5, 52.4] | - |
| **Season**  non-heating (ref: heating) | -62.8 [-71.8, -50.9]*** | -67.7 [-77.6, -53.5]*** | -55.0 [-71.0, -30.3]*** | - | - |
| **Outdoor concentrations**  **per 10 μg/m^3^*** | 5.8 [4.7, 6.9]*** | 6.0 [4.6, 7.4]*** | 5.5 [3.8, 7.1]*** | 3.5 [2.0, 5.1]*** | 9.8 [8.0, 11.6]*** |
| **Ambient relative humidity, per %** | 0.8 [0.5, 1.1]*** | 0.8 [0.4, 1.2]*** | 0.7 [0.3, 1.1]*** | 1.5 [1.1, 2.0]*** | 0.8 [0.3, 1.2]*** |
| **Ambient temperature, per °C** | 3.4 [2.2, 4.6]*** | 4.3 [2.7, 5.9]*** | 2.1 [0.3, 4.0]** | 6.0 [3.1, 8.9]*** | 1.9 [0.7, 3.1]*** |
| **Site**  Guangxi (ref)  Beijing  Shanxi | 37.9 [15.0, 65.3]***  69.9 [43.1, 101.6]*** | 45.1 [14.3, 84.2]***  71.8 [36.7, 115.9]*** | 26.8 [-4.9, 69.2]  65.7 [26.7, 116.7]*** | (ref)  -25.6 [-37.5, -11.5]*** | 5.5 [-8.8, 22.1]  120.9 [89.3, 157.7]*** |
| **Marginal R^2^** | 0.24 | 0.23 | 0.25 | 0.25 | 0.25 |
| **Conditional R^2^** | 0.29 | 0.29 | 0.33 | 0.38 | 0.35 |

**Table S3. Associations between personal PM_2.5_ exposure and selected sociodemographic, energy use, and environmental variables by gender and season, expressed as % change in exposure [95% CI]^a^**

*p-value <0.10; **p-value <0.05; ***p-value<0.001

^a^ Regression of log-air pollution exposure can be converted to the percent (%) change in exposure using the equation ([exp^β^ – 1]x 100), where β is the change in log-transformed pollution exposure associated with a one-unit change in the independent variable.

| **N_participants_ (N_filters_)** | **Main analysis PM_2.5_ model (all observations)**  **746 (2022)** | **(1) Analysis limited to PM_2.5_ exposure samples within ±10% of 24-h target**  **744 (1913)** | **(2) Analysis limited to PM_2.5_ exposure samples that could be matched with village-level outdoor PM_2.5_ measurements**  **(village-level outdoor PM_2.5_)**  **418 (606)** | **(3) Analysis limited to PM_2.5_ exposure samples that could be matched with village-level outdoor PM_2.5_ measurements**  **(Outdoor PM_2.5_ estimated from government monitors)**  **418 (606)** | **(4) Analysis limited to PM_2.5_ exposure samples with no potentially non-compliant samples**  **(<500 steps)**  **742 (1978)** |
| --- | --- | --- | --- | --- | --- |
| **Age per year** | -0.2 [-0.8,0.4] | 0.0 [-0.6, 0.6] | 0.1 [-0.9, 1.2] | 0.2 [-0.9, 1.3] | -0.2 [-0.8, 0.4] |
| **Gender**  male (ref: female) | 4.6 [-6.1, 16.5] | 3.3 [-7.2, 15.0] | 21.4 [-0.5, 48.2]* | 19.8 [-1.7, 46.0]* | 5.6 [-5.2, 17.6] |
| **Occupation**  agriculture (ref)  other work outside the home  not working outside the home | 5.9 [-10.3, 24.9]  -3.2 [-13.1, 7.8] | 7.6 [-8.6, 26.8]  -4.6 [-14.4, 6.2] | -7.1 [-28,1, 20.0]  -1.0 [-17.8, 19.2] | -7.5 [-28.2, 19.3]  -2.8 [-19.1, 16.9] | 7.4 [-8.9, 26.8]  -1.3 [-11.4, 10] |
| **Annual household income (yuan)**  <20000 (ref: ≥20000) | 3.1 [-5.9, 13.0] | 2.3 [-6.6, 12.1] | -0.5 [-15.2, 16.8] | -1.5 [-16.0, 15.4] | 3.4 [-5.6, 13.4] |
| **Education**  college/high (ref)  primary  no school | 0.4 [-8.7, 10.5]  -5.4 [-17.2, 8.1] | 0.5 [-8.6, 10.5]  -6.2 [-17.7, 7.0] | -8.0 [-22.6, 9.3]  -13.7 [-31.2, 8.2] | -8.8 [-23.2, 8.3]  -13.6 [-31.0, 8.2] | 2.0 [-7.3, 12.1]  -6.0 [-17.7, 7.4] |
| **Smoker**  smoker (ref)  non-smoker w/ household smoker  non-smoker w/o household smoker | -26.2 [-36.3, -14.4]***  -30.4 [-38.0, -21.8]*** | -27.2 [-37.2, -15.6]***  -32.4 [-39.7, -24.1]*** | -3.4 [-26.0, 26.2]  -28.1 [-41.3, -11.8]*** | -3.9 [-26.3, 25.3]  -28.2 [-41.4, -12.1]*** | -27.2 [-37.2, -15.5]***  -31.7 [-39.1, -23.3]*** |
| **Cooking fuel**  clean fuel use (ref:solid fuel) | -15.4 [-22.3, -8.0]*** | -15.0 [-21.8, -7.5]*** | -1.1 [-14.8, 14.8] | -1.9 [-15.5, 13.8] | -15.4 [-22.3, -8.0]*** |
| **Heating fuel**  indoor solid fuel (ref)  outdoor solid fuel use  only clean fuel  no devices | -24.6 [-36.9, -9.8]***  -2.8 [-12.3, 7.7]  -1.6 [-16.6, 16.3] | -24.2 [-36.5, -9.6]***  0.1 [-9.6, 10.8]  0.1 [-15.2, 18.1] | -10.5 [-36.7, 26.6]  -1.1 [-17.9, 19.0]*  4.9 [-21.1, 39.4] | -11.1 [-37.0, 25.4]  -4.1 [-20.3, 15.5]*  2.3 [-23.0, 35.8] | -26.1 [-38.3, -11.5]***  -1.9 [-11.4, 8.7]  2.0 [-13.7, 20.5] |
| **Season^b^**  non-heating (ref:heating) | -62.8 [-71.8, -50.9]*** | -62.3 [-71.4, -50.4]*** | - | - | -63.2 [-72.1, -51.4]*** |
| **Outdoor concentrations PM_2.5_ per 10 μg/m^3^*** | 5.8 [4.7, 6.9]*** | 5.7 [4.6, 6.8]*** | 3.4 [1.7, 5.0]*** | 2.4 [1.3, 3.5]*** | 5.7 [4.7, 6.9]*** |
| **Ambient RH per %** | 0.8 [0.5, 1.1]*** | 0.8 [0.5, 1.1]*** | 1.0 [0.4, 1.6]*** | 0.7 [0.0, 1.4]*** | 0.8 [0.5, 1.1]*** |
| **Ambient temperature per °C** | 3.4 [2.2, 4.6]*** | 3.4 [2.2, 4.6]*** | 2.2 [-1.0, 5.5] | 2.4 [-0.7, 5.7] | 3.5 [2.3, 4.7]*** |
| **Site**  Guangxi (ref)  Beijing  Shanxi | 37.9 [15.0, 65.3]***  69.9 [43.1, 101.6]*** | 42.2 [18.7, 70.3]***  71.8 [44.8, 103.9]*** | 293.4 [103.1, 662.2]***  208.3 [66.3, 471.7]*** | 254.6 [81.6, 592.7]***  187.7 [54.7, 435.0]*** | 46.2 [22, 75.4]***  78.7 [50.5, 112.2]*** |
| **Marginal R^2^** | 0.24 | 0.25 | 0.31 | 0.31 | 0.25 |
| **Conditional R^2^** | 0.29 | 0.30 | 0.46 | 0.46 | 0.31 |

**Table S4. Sensitivity analysis of associations between personal PM_2.5_ exposure and selected sociodemographic, energy use, and environmental variables, expressed as % change in exposure [95% CI]^a^**

*p-value <0.10; **p-value <0.05; ***p-value<0.001

^a^ Regression of log-air pollution exposure can be converted to the percent (%) change in exposure using the equation ([exp^β^ – 1]x 100), where β is the change in log-transformed pollution exposure associated with a one-unit change in the independent variable.
